# Motivation Predicts Change in Nurses’ Physical Activity Levels During a Web-Based Worksite Intervention: Results From a Randomized Trial

**DOI:** 10.2196/11543

**Published:** 2020-09-11

**Authors:** Jennifer Brunet, Heather E Tulloch, Emily Wolfe Phillips, Robert D Reid, Andrew L Pipe, Jennifer L Reed

**Affiliations:** 1 Faculty of Health Sciences University of Ottawa Ottawa, ON Canada; 2 Institut du savoir de l’Hôpital Montfort Hôpital Montfort Ottawa, ON Canada; 3 Cancer Therapeutic Program Ottawa Hospital Research Institute Ottawa, ON Canada; 4 Division of Prevention and Rehabilitation University of Ottawa Heart Institute Ottawa, ON Canada; 5 Faculty of Medicine University of Ottawa Ottawa, ON Canada

**Keywords:** physical activity, motivation, wearable technology, nurses

## Abstract

**Background:**

Low physical activity levels can negatively affect the health of nurses. Given the low physical activity levels reported by nurses, there is a clear need for brief and economical interventions designed to increase physical activity levels in this population. We developed a web-based intervention that used motivational strategies to increase nurses’ physical activity levels. The intervention provided the nurses with feedback from an activity monitor coupled with a web-based individual, friend, or team physical activity challenge.

**Objective:**

In this parallel-group randomized trial, we examine whether nurses’ motivation at baseline predicted changes in objectively measured physical activity levels during the 6-week intervention.

**Methods:**

The participants were 76 nurses (n=74, 97% female; mean age 46, SD 11 years) randomly assigned to 1 of 3 physical activity challenge conditions: (1) individual, (2) friend, or (3) team. The nurses completed a web-based questionnaire designed to assess motivational regulations for physical activity levels before the intervention and wore a Tractivity activity monitor before and during the 6-week intervention. We analyzed data using multilevel modeling for repeated measures.

**Results:**

The nurses’ physical activity levels increased (linear estimate=10.30, SE 3.15; *P*=.001), but the rate of change decreased over time (quadratic estimate=−2.06, SE 0.52; *P*<.001). External and identified regulations (ß=−2.08 to 11.55; *P*=.02 to .04), but not intrinsic and introjected regulations (ß=−.91 to 6.29; *P*=.06 to .36), predicted changes in the nurses’ physical activity levels.

**Conclusions:**

Our findings provide evidence that an intervention that incorporates self-monitoring and physical activity challenges can be generally effective in increasing nurses’ physical activity levels in the short term. They also suggest that drawing solely on organismic integration theory to predict changes in physical activity levels among the nurses participating in web-based worksite interventions may have been insufficient. Future research should examine additional personal (eg, self-efficacy) and occupational factors (eg, shift length and shift type) that influence physical activity levels to identify potential targets for intervention among nurses.

**Trial Registration:**

ClinicalTrials.gov NCT04524572; https://clinicaltrials.gov/ct2/show/NCT04524572

## Introduction

### Background

Engaging in regular physical activity can improve cardiovascular function and musculoskeletal strength, reduce the risk of morbidity and mortality due to chronic disease, and decrease the risk of mental health problems such as anxiety and depression [1-3]. In addition, engaging in physical activity can reduce work-related stress and the incidence of burnout [4-7]—a major problem for health care workers [8]. Many investigators have shown that nurses, who represent 48% of the health care workforce [9], report high levels of work-related stress and burnout, low levels of job satisfaction, and poor health [10-14]. Despite the known benefits of physical activity, nurses’ physical activity levels remain low [15-18]. Common barriers to physical activity reported by nurses include busy schedules, irregular shifts, long hours, and a lack of time, suggesting that the worksite may be an ideal place to intervene to increase nurses’ physical activity levels [19]. Beyond personal health benefits, worksite interventions seeking to increase nurses’ physical activity levels have the potential to improve employee performance, lower employee health care costs, and decrease absenteeism rates, which are higher in nurses than in other occupational groups [20].

The internet is a promising way to deliver worksite interventions, as it affords timely access and the ability to reach a larger population [21]. It may be particularly appropriate for nurses whose long working hours and irregular shifts preclude opportunities to participate in traditional face-to-face interventions that are often scheduled to accommodate those with relatively fixed schedules. There is mounting evidence that web-based interventions can help increase physical activity levels among working adults [22-25]. Nevertheless, some web-based worksite interventions have not led to significant increases in physical activity levels [26,27].

Although it has been recognized that changes in behaviors associated with a particular intervention may be influenced by the personal characteristics of the participants, few researchers evaluating the effects of web-based worksite interventions have sought to identify which characteristics, apart from sociodemographic factors, have an influence on behavior change [26,28-32]. Consequently, there is limited knowledge of other factors that may predict physical activity levels among web-based worksite intervention participants. An examination of additional factors that might predict physical activity levels in web-based worksite interventions is critical to acquire an enhanced understanding of the forces that impel change. There is robust evidence that motivation is a strong predictor of participation in physical activity [33]; researchers might, therefore, consider drawing on motivational theories such as the organismic integration theory [34,35]—1 of the 6 mini theories of self-determination theory—to ascertain whether motivation predicts physical activity levels among web-based worksite intervention participants.

### Objectives

To address the aforementioned gaps in the literature, we developed a web-based worksite intervention for nurses working in a tertiary care cardiovascular institute. We created individual, friend, and team challenge groups in which the nurses would track their physical activity levels using a Tractivity activity monitor and upload their activity data at times and frequencies of their choosing because of the effectiveness of self-monitoring [36]. The nurses randomized to the friend and team challenge groups would also share their physical activity levels in deidentified format with one other nurse (friend challenge group) or a team of nurses (team challenge group) randomly chosen, which was presumed to motivate them to be more active to make a positive impression on members of their group according to self-presentation perspectives [37,38]. Furthermore, based on the principles of the social comparison theory [39], it was presumed that allowing the nurses randomized to the friend and team challenge groups to exchange physical activity level data would serve as a basis for social comparison, and such comparisons would impel further behavior change. For example, social comparisons could allow the nurses to develop an internal norm of what a *good* physical activity level is and encourage them to adjust their levels if there was a discrepancy. In this regard, observing better-performing nurses would prompt the nurses to increase their physical activity levels to reduce the discrepancy between themselves and others to make themselves feel good about their current levels.

Using data collected as part of a trial evaluating changes in physical activity levels and the impact on cardiovascular risk factors among nurses participating in a web-based worksite intervention [40], we examined whether the nurses’ motivation predicted changes in their objectively measured daily physical activity levels. Using the organismic integration theory [34], we assessed 5 core motivational *regulations*: intrinsic motivation (ie, a person pursues an activity for the inherent pleasure and enjoyment of the activity), identified regulation (ie, a person pursues an activity that they deem personally valuable and important to attain a desired outcome), introjected regulation (ie, a person pursues an activity to avoid feelings of guilt and shame and/or protect feelings of worth and ego), external regulation (ie, a person pursues an activity because of external demands, eg, punishments, threats, and/or possible rewards), and amotivation (ie, a person has a relative absence of intrinsic or extrinsic motivation and lacks a reason to act). On the basis of the organismic integration theory [34] and past research [33,41], we hypothesized that self-determined motivational regulations (ie, intrinsic motivation and identified regulation) would positively predict initial levels of and changes in objectively measured daily physical activity levels among the study participants. As researchers have observed inconsistent associations between non–self-determined motivational regulations (ie, introjected regulation, external regulation, and amotivation) and physical activity levels [33,41], we further hypothesized that these regulations would be unrelated or negatively associated with initial levels of and changes in objectively measured daily physical activity levels.

## Methods

### Setting and Procedures

Following ethics approval by the University of Ottawa Heart Institute Research Ethics Board (Protocol No. 20130429), nurses working at the University of Ottawa Heart Institute—a tertiary care cardiovascular institute—were recruited to participate in this parallel-group randomized trial. Further details about the study design and procedures have been reported previously [40]. Briefly, recruitment took place between September 2013 and November 2013 via posters distributed throughout the University of Ottawa Heart Institute, word of mouth, and announcements during nursing meetings and morning rounds. The nurses were eligible if they were (1) a registered nurse, (2) able to walk unassisted, (3) willing to wear a stretchable ankle band that contained a physical activity monitoring device (ie, accelerometer) and had access to the internet, and (4) able and willing to provide written informed consent. The nurses were not eligible if they were (1) pregnant or lactating, (2) unable to read and understand English, (3) having medical contraindications to exercise, and/or (4) already using an activity monitor to track their physical activity levels. Nurses who were interested and believed they were eligible were invited to contact the study staff who confirmed final eligibility.

Once eligibility was confirmed, the nurses attended a study enrollment session with study staff where they provided written informed consent and then received a Tractivity activity monitor along with instructions for using it and instructions for logging onto and uploading data to their Tractivity web account (Multimedia Appendix 1). The nurses were instructed to wear the activity monitor from waking to bedtime (except during water activities) throughout the baseline (1 week) and intervention (6 weeks) phases. In addition, they were asked to complete self-report measures (eg, sociodemographics, work-related characteristics, and motivational regulations for physical activity) at baseline and had their resting blood pressure, heart rate, and anthropometric measurements (ie, height, body mass, waist circumference, and body fat percentage) taken by research staff who were blinded to the assigned groups of the participants. Further details regarding these assessments can be found in a study by Reed et al [40].

### Participants

**Figure 1:**
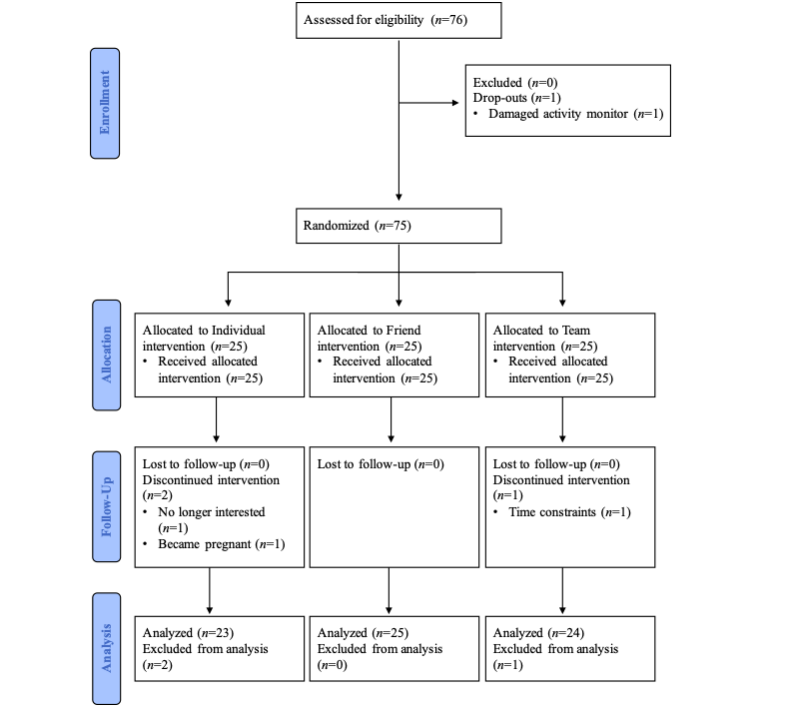
Consolidated Standards of Reporting Trials (CONSORT) flow diagram of nurses recruited and reasons for withdrawals.

In total, 76 nurses contacted the research staff, met eligibility criteria, and consented to participate in this study (Figure 1). Their mean age was 46.3 (SD 10.9) years, mean BMI was 27.5 (SD 5.6) kg/m^2^, and their mean resting blood pressure was 115 (SD 12)/75 (SD 8) mm Hg. On the basis of these values, they were categorized as being mostly overweight and normotensive. Most were female (74/76, 97%), worked only day shifts (40/76, 53%), and performed clinical duties (53/76, 70%). Only 3 of the 76 (4%) participants met the current physical activity guidelines at baseline. Additional information describing the participants’ demographics, anthropometrics, shift profiles, and nursing roles are presented by Reed et al [40] (Table 1).

**Table 1:** Fixed effects and fit statistics for the multilevel growth models of moderate-to-vigorous physical activity.

Variables	Model 1		Model 2		Model 3		Model 4	
	Estimate (SE)	*P* value	Estimate (SE)	*P* value	Estimate (SE)	*P* value	Estimate (SE)	*P* value
Intercept	44.43 (5.13)	<.001	34.71 (4.63)	<.001	30.49 (12.24)	.02	34.85 (4.45)	<.001
Time	−1.96 (0.79)	.02	10.30 (3.15)	.002	15.04 (8.37)	.08	10.26 (3.03)	.001
Time squared	—^a^	—	−2.06 (0.52)	<.001	−2.83 (1.38)	.04	−2.05 (0.50)	<.001
Group	—	—	—	—	2.14 (5.74)	.71	—	—
Group×time	—	—	—	—	−2.40 (3.92)	.54	—	—
Group×time squared	—	—	—	—	0.39 (0.65)	.55	—	—
External	—	—	—	—	—	—	−1.60 (7.11)	.82
Introjected	—	—	—	—	—	—	5.65 (4.82)	.24
Identified	—	—	—	—	—	—	11.43 (7.75)	.14
Intrinsic	—	—	—	—	—	—	1.51 (6.33)	.81
External×time	—	—	—	—	—	—	11.55 (4.83)	.02
Introjected×time	—	—	—	—	—	—	−6.29 (3.27)	.06
Identified×time	—	—	—	—	—	—	11.38 (5.35)	.04
Intrinsic×time	—	—	—	—	—	—	−4.05 (4.37)	.36
External×time squared	—	—	—	—	—	—	−1.87 (0.80)	.02
Introjected×time squared	—	—	—	—	—	—	0.91 (0.54)	.10
Identified×time squared	—	—	—	—	—	—	−2.08 (0.89)	.02
Intrinsic×time squared	—	—	—	—	—	—	0.75 (0.73)	.31
2 restricted log likelihood	4803.96	N/A^b^	4747.55	N/A	4739.56	N/A	4696.33	N/A
Akaike information criterion	4811.96	N/A	4761.55	N/A	4753.56	N/A	4710.33	N/A
Schwarz Bayesian information criterion	4828.61	N/A	4790.68	N/A	4782.65	N/A	4739.28	N/A

^a^There are no results to report.

^b^N/A: not applicable.

### Randomization and Intervention Groups

Of the 76 nurses who provided consent, 75 (99%) were randomized to the individual, friend, or team physical activity challenge groups, and 1 (1%) dropped out following the baseline assessment because of a damaged Tractivity activity monitor (Figure 1). Randomization to the 3 groups was conducted by research staff using the *RAND* function of a software spreadsheet program (Microsoft Excel) in a 1:1:1 ratio. The participants were notified of their assigned group via email. In the individual challenge group, the participants were able to log onto their Tractivity web account at any time during the intervention phase to track their physical activity levels (ie, distance [km], steps [counts], active time [min], and calories [kcal]) displayed in a graphical format in the web-based Tractivity program. In the friend and team challenge groups, the participants were also able to log onto their Tractivity web account at any time during the intervention phase to track their own physical activity levels, but they could also monitor the physical activity levels of either one other participant (friend challenge group; Multimedia Appendix 2) or 4 other participants (team challenge group; Multimedia Appendix 3). The participants in the friend and team challenge groups were blinded in keeping with ethical considerations; none knew the identity of the other participant or team members in their group.

### Study Assessments

Motivational regulations for physical activity were assessed at baseline using the 19-item Behavioral Regulation in Exercise Questionnaire-2 (BREQ-2) [42]. The participants were presented with the stem, “Using the scale below, please indicate to what extent each of the following items is true for you,” followed by items representing amotivation (4 items; eg, “I can’t see why I should bother exercising”), external (4 items; eg, “I feel under pressure from my friends/family to exercise”), introjected (3 items; eg, “I feel ashamed when I miss an exercise session”), identified (4 items; eg, “I value the benefits of exercise”), and intrinsic (4 items; eg, “I exercise because it’s fun”) regulations. Items were rated on a 5-point Likert scale, ranging from 0 (*not true for me*) to 4 (*very true for me*). Integrated regulation is not assessed on this scale because it is difficult to differentiate between integrated and identified regulation [43]. We calculated subscale scores by averaging responses of items belonging to the same subscale; however, only the external, introjected, identified, and intrinsic regulation subscales were analyzed in this study because of the extremely low variance and the high number of zeros for the amotivation subscale. The reliability and validity of BREQ-2 scores have been previously demonstrated [44,45].

Physical activity was measured regularly during the baseline (1 week) and intervention (6 weeks) phases using the Tractivity activity monitor), which is a lightweight, compact accelerometer that uses a proprietary signal processing algorithm to determine step counts in 1-min intervals. The activity monitor provides no visible feedback and stores up to 30 days of data (ie, distance, steps, active time, and calories). Research staff uploaded the participants’ activity data into the web-based Tractivity program at the end of the baseline and intervention phases. The participants uploaded their activity data at times and frequencies of their choice throughout the intervention phase. The Tractivity activity monitor has been shown to be a valid measure of step counts in comparison with direct observation [46]. Activity monitors were calibrated for stride length before the baseline week by having the participants walk 10 steps (at their usual walking speed) in a straight line on a large indoor track. These measures were performed in triplicate, and the average was entered into the web-based Tractivity program to assist the proprietary signal processing algorithm in calculating step counts.

The monitors provided us with consecutively ordered min-by-min activity data (ie, steps [counts], distance [km], active time [min], and calories [kcal]) during each day of the baseline and intervention phases for all the participants. We used a Hypertext Preprocessor (PHP, version 7.0) script to process the data. All activity monitor data were screened to identify valid and nonvalid days. Data were considered valid and included in the analysis if the wear time was at least 10 hours [47]. Step counts were used to calculate min of moderate-to-vigorous physical activity (MVPA) levels in bouts of at least 10 min [48,49]. Previously established cut-points (ie, >100 steps per min) [50] were used to calculate daily min of MVPA.

### Sample Size

A post hoc power analysis revealed that the sample size of 76 participants provided adequate power (1−β=.92) to detect significant differences in physical activity levels within and between groups of small magnitude (ie, eta-squared value of 0.022 with an α of .05).

### Statistical Analysis

All data analyses were performed using SPSS (version 24; IBM Corp), and *P*<.05 was considered statistically significant. Descriptive characteristics of the study sample were summarized using mean (SD) or frequencies (%). Data were analyzed using multilevel growth modeling as repeated observations were nested within the participants who were nested within the groups [51]. When analyzing longitudinal data, multilevel growth modeling also offers the following advantages: (1) equally spaced periods are not required, (2) the number of time points may vary across the participants, allowing for the use of data from all the participants to provide unbiased estimates of the outcomes, assuming data are missing at random, and (3) missing data are not problematic as long as they are missing at random [52]. Before these analyses, a 2-step approach for transforming continuous, nonnormalized variables to normal variables was applied to the MVPA data [53], as preliminary analysis revealed that the MVPA data were not normally distributed.

We then estimated an unconditional multilevel linear growth model for MVPA (model 1) and compared it with an unconditional quadratic growth model (model 2) to formally test the optimal functional form of growth. In doing so, we created a new variable *time*, for which baseline was coded as *0* to serve as the reference point, and subsequent time points were assigned the following values: 1, 2, 3, 4, 5, and 6. This coding accounts for any differences in time intervals between points and allowed for the interpretation of the intercept as predicted MVPA levels at baseline. In addition, fixed and random effects for time were included because it was assumed that not all the participants had the same baseline MVPA levels or the same exact rate of change over time. The fixed effects provide estimates of the average levels at baseline and average rate of change for the sample, whereas the random effects serve to ascertain whether there is variability in baseline levels and in the rate of change. These competing models were compared using a likelihood ratio test and 2 commonly used information criteria, namely, Akaike information criterion and Schwarz Bayesian information criterion. The model that minimized Akaike information criterion and Bayesian information criterion values was retained.

Next, we expanded the retained unconditional growth model by adding group, group by time, and group by time squared as predictors of MVPA to test the effect of group (model 3). There was no statistically significant main effect for group (*P*=.71) or interaction between time (and time square) and group (Table 1, model 3; *P*=.54 to .55). This demonstrated that there were no group differences in MVPA levels at baseline or in change over time. We calculated the intraclass correlation coefficient to further assess dependence in the grouped data. As the coefficient was less 0.05 (indicating that dependence related to group membership could be ignored) [54], we proceeded to test subsequent models without group, group by time, and group by time squared.

Finally, we added the motivational regulations as predictors to the unconditional quadratic growth model to test the effect of each regulation on MVPA (model 4). To fit this conditional growth model, we added the main effects of each regulation along with their interaction with time (and time squared). Of note, each regulation was grand-mean centered by subtracting the sample mean from each observed value to make the interpretation of the model parameters easier.

## Results

### Participants

Of the 75 participants randomized, 72 (96%) completed all study assessments, including 92% (23/25) assigned to the individual challenge, 100% (25/25) assigned to the friend challenge, and 96% (24/25) assigned to the team challenge. A one-way analysis of variance was performed to compare baseline MVPA levels between the participants who dropped out and those who completed the 6-week intervention; results revealed no significant differences in baseline MVPA levels.

### Main Results

**Figure 2:**
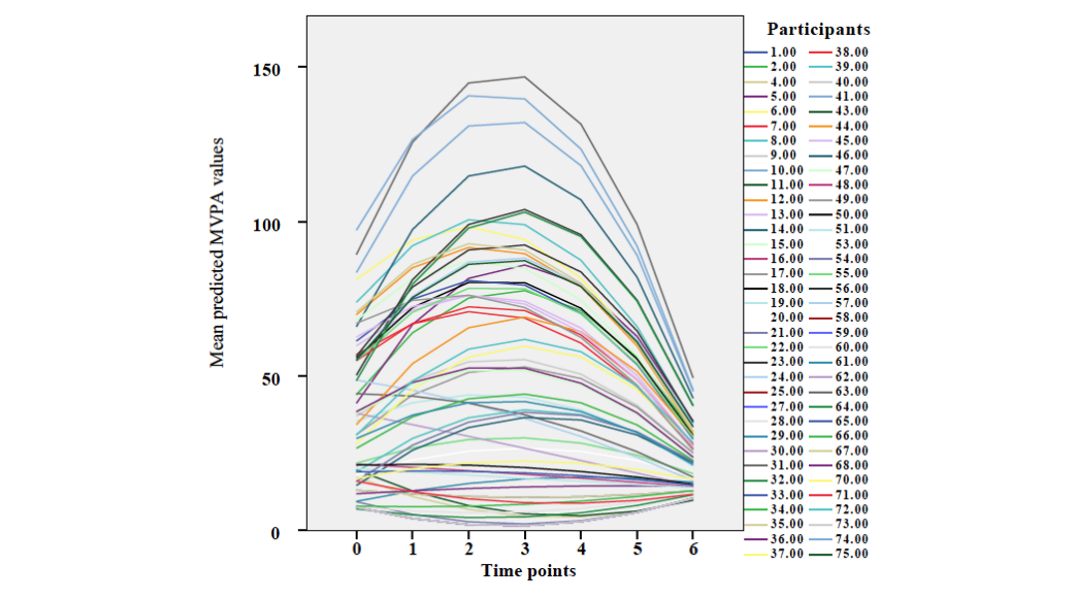
A plot illustrating the individual trajectories for MVPA levels at baseline (week 0) and throughout the intervention phase (weeks 1-6). More negative slopes correspond to greater decreases in MVPA levels. MVPA: moderate-to-vigorous physical activity.

Visual inspection of the plotted trajectories using predicted normalized MVPA values (Figure 2) suggested that it may not be adequate to summarize the pattern of change over time with a linear trajectory, but rather a quadratic trajectory over time. In addition, there were several indications that a quadratic growth model was the most appropriate for representing the individual growth trajectories of MVPA levels (Table 1, models 1 and 2). First, the Akaike information criterion and Bayesian information criterion values were smaller for the quadratic growth model. Second, the fixed quadratic effect and variance components of the quadratic model were significant and of nontrivial magnitude. Third, after refitting the 2 models with full information maximum likelihood, a likelihood ratio test comparing the linear model with the quadratic model indicated that the former should be rejected in favor of the latter. The fixed effects were significant in the quadratic unconditional growth model (Table 1, model 2), demonstrating that the mean MVPA baseline level was 34.71 (SE 4.63; *P*<.001) min per week and that levels changed significantly over time in a curvilinear (ie, inverted U shape) fashion (linear estimate=10.30, SE 3.15; *P*=.002; and quadratic estimate=−2.06, SE 0.52; *P*<.001). In addition, the random effects for (1) the intercept (estimate=815.85, SE 258.40; *P*=.002), (2) the slopes for MVPA (linear estimate=269.39, SE 122.85; *P*=.03; and quadratic estimate=8.07, SE 3.47; *P*=.02), and (3) the covariance between the intercepts and quadratic slopes (estimate=−50.86, SE 21.22; *P*=.02) were significant. These findings demonstrate that there was meaningful variability in (1) MVPA levels at baseline between the participants, and (2) changes in MVPA levels over time. Furthermore, the participants who engaged in more MVPA at baseline tended to have greater increases in MVPA levels initially, followed by steeper decreases.

The results of the conditional quadratic growth model, in which we added the main effects of each grand-mean centered motivational regulation along with their interaction with time (and time squared), are presented in Table 1 (model 4). No significant main or interaction effects were observed for introjected regulation or intrinsic motivation (*P*=.06 to .81). In contrast, there were significant effects for external and identified regulations. Specifically, there was a significant interaction between time (and time squared) and external regulation (*P*=.02) as well as between time (and time squared) and identified regulation (*P*=.02 to .04). These findings indicate that there are differences in the rate of change in MVPA levels as a function of the external and identified regulations levels of the participants at baseline. To better understand the nature of these relationships, we probed both interactions by a test of simple slopes at specific values of external and identified regulations, namely, at high (1 SD above the mean), medium (at the mean), and low (1 SD below the mean) levels of each regulation [55,56]. Probing showed that initial increases in MVPA levels were significant for the participants reporting medium (estimate=−3.93, SE 1.58; *P*=.02) and high (estimate=−5.70, SE 2.49; *P*=.03) levels of external regulation at baseline, but not for those with low levels of external regulation (estimate=−2.16, SE 1.12; *P*=.06). Similarly, probing showed that initial increases in MVPA levels were significant for the participants reporting medium (estimate=−3.96, SE 1.57; *P*=.01) and high (estimate=−6.76, SE 2.48; *P*=.009) levels of identified regulation at baseline, but not for those with low levels of identified regulation (estimate=−1.16, SE 1.10; *P*=.30).

## Discussion

### Principal Findings

Given the importance of identifying factors that may help to promote changes in physical activity levels within the workplace setting, the aim of this study was to examine whether motivation predicted changes in objectively measured daily physical activity levels among participating nurses. Our principal finding is that some, namely, external and identified regulations, but not all types of motivation, predicted changes in the nurses’ physical activity levels throughout the intervention. According to the World Health Organization, the workplace is an ideal setting to implement health promotion initiatives to reduce noncommunicable disease risk factors [57]. Although worksite interventions seeking to increase physical activity levels among health care workers (eg, allied health care providers and administrative staff) have been developed and implemented, few have targeted nurses specifically. Only half of these interventions significantly increased physical activity levels (eg, steps, daily min, and energy expenditure) [58]. Nurses differ from other hospital workers because they may work long shifts (ie, 8- to 12-hour shifts), irregular hours (ie, rotating day, evening, or night shifts), and undertake physically demanding tasks (eg, transfer patients between beds, chairs, and wheelchairs, reposition patients, push or pull beds, chairs, and wheelchairs, and carry equipment) [59], all of which can adversely affect their health. The traditional mode of delivering worksite interventions is face-to-face, but worksite and job characteristics may hinder nurses’ ability to participate in such programs. Recognizing that nurses report low physical activity levels [60]—a known risk factor for the onset of noncommunicable diseases [61] and that the internet may offer a way to reach nurses—we developed and implemented a web-based worksite intervention for nurses working in a tertiary care cardiovascular institute.

Compared to nurses with low levels of external regulation at baseline, the nurses with medium and high levels of external regulation at baseline had greater increases in MVPA levels at the start of the intervention phase in this study. Contrary to the belief that external incentives can decrease people’s motivation to participate in physical activity [35,62,63], these findings suggest that external incentives (eg, financial incentives, competition prizes, and recognition from others), pressures, and sanctions may play a role in initially increasing physical activity levels. However, the effects of external incentives appear to be beneficial only in the short term, as the nurses’ physical activity levels were not maintained at the end of the intervention phase (Figure 2). As previously observed by other researchers [62,63], external incentives, pressures, and sanctions may undermine people’s self-determined motivation to participate in physical activity, which raises questions concerning the use of such strategies to help nurses *maintain* physical activity levels over time. More research is needed to understand whether and when external incentives, pressures, and sanctions could be used to increase nurses’ physical activity levels. It is possible that they help nurses who are not regularly active commence activities until they recognize and enjoy the intrinsic rewards that accompany physical activity (eg, healthy weight, better sleep, stress management, and improved psychological health).

Motivation has been shown to positively influence physical activity behavior when pursuing an activity that is deemed personally valuable and in which it is important to attain a desired outcome [33]. In support of these findings, the nurses in this study who possessed medium and high levels of identified regulation at baseline tended to have greater initial increases in MVPA. This suggests that it is necessary to help nurses recognize and enjoy the physical, psychological, and social benefits that accompany physical activity to help them increase their physical activity levels [33]. However, this approach may not be sufficient long-term; the findings of this study also showed that the nurses with medium and high levels of identified regulation had greater decreases in physical activity levels at the end of the intervention phase. There are several factors that may have interfered with the nurses’ ability to maintain physical activity levels over time. On a personal level, nurses have often identified barriers to physical activity such as high workloads, conflicting schedules, and physical and emotional stress in their workplace. These barriers may have interfered with the nurses’ ability to maintain physical activity levels in this study. It is also possible that, despite being motivated, the nurses lacked confidence and skills to sustain high physical activity levels. To test this hypothesis, interventions seeking to enhance nurses’ physical activity confidence and skills by providing teaching, training, and/or counseling on goal setting, self-monitoring, and action planning should be developed and evaluated to determine if this leads to sustained changes over a longer period [64-66]. Furthermore, drawing on evidence-based behavior change techniques [36], providing: (1) coaching, (2) social support from family, friends, and staff, (3) feedback on progress and barrier identification or problem solving, (4) follow-up prompts, and (5) health checks may help to reinforce long-term changes in physical activity.

In addition to the personal factors that may have hindered sustained physical activity change in this study, targeting the entire worksite environment as opposed to the nurses within it might have increased the effectiveness of our intervention, as occupational constraints may have further inhibited the nurses’ ability to maintain physical activity levels. Speculatively, worksite characteristics such as management structure, leadership, culture, and support for physical activity within the workplace may have been insufficient for the nurses in this study to translate their intention into long-term physical activity changes. Accordingly, comprehensive interventions that target both personal (eg, motivation and self-management) and macro-level factors (eg, worksite environment) may be warranted. With regard to the latter, policy interventions (eg, arranging physical activity breaks during work) or environmental changes (eg, using physical activity level prompts in common areas [break rooms, bathrooms, and elevators or stairwells], forming lunchtime physical activity groups, promoting stairway signs, and having indoor and outdoor walking routes) may help to promote sustained physical activity.

Fostering social support in the workplace has been shown to be an effective way to increase physical activity levels [66,67]. In this study, the nurses were randomized to individual, friend, or group challenges; however, no significant differences in initial levels of or changes in physical activity levels were observed between the groups. As the identities of the friend or group members were not disclosed to the nurses, it is possible that an opportunity to foster social support was missed. Future interventionists should consider permitting the participants to know the identity of their fellow participants and facilitate social support and relatedness (rather than simply social comparison) among them. Indeed, within basic psychological needs theory [34]—another mini theory of self-determination theory—perceived relatedness (ie, experience of belongingness and connectedness to others), as well as perceived competence (ie, feelings of effectiveness and ability to achieve desired outcomes) and autonomy (ie, experience of self-determination and volition when carrying out an activity), has been identified as necessary for promoting adaptive behavioral outcomes [34,35]. Several studies indicate that individuals benefit from feeling connected to others [33].

### Limitations

Although promising, the results of this study should be interpreted with caution. First, this study was conducted at a single worksite, a tertiary care cardiovascular institute, and the sample size was relatively small. Although there are similarities in some nursing roles, the local context may impact nurses’ physical activity levels as a result of differing systems of nursing care, facilities, patient load, and resources. The generalizability of the findings of this study to nurses working in other health care settings and systems merits further exploration with larger sample sizes. Motivational regulations were only assessed at baseline. Thus, it is not clear to what extent the intervention impacted the nurses’ motivation over time and to what extent this was related to changes in their physical activity levels. For example, the nurses’ motivation may have increased or waned when comparing their activity level with others or as they gained more experience and confidence exercising over the 6-week intervention. There is a risk that the present findings reflect a selection bias as the participants were self-selecting. It is possible that the nurses who participated in this study may only be those who felt that they were healthy and fit enough to engage in a physical activity intervention and valued such activity. The nonsignificant associations between certain motivational regulations and changes in physical activity levels may be explained by the fact that the nurses in this study had relatively low (or high) scores at baseline, which may have precluded the ability to detect significant associations because of the limited variability in scores. There was some attrition, although dropout was distributed evenly across the groups. Finally, the intervention was only 6 weeks long, which may not have permitted time to facilitate long-term physical activity change.

### Implications for Future Research and Practice

Understanding how best to promote physical activity among nurses remains an important endeavor, as their low physical activity levels suggest that they are at increased risk of chronic diseases and, consequently, are at higher risk of being absent from work. To allow for a better consideration of the potential impact of interventions on nurses’ physical activity levels, more nurse-only intervention studies drawing on theories from the fields of psychology, sociology, behavioral economics, and/or management are needed to identify personal, situational, environmental, structural, and lifestyle factors that influence participation and effectiveness. For example, as the nurses randomized to the friend or team challenge groups might have formed expectations of how much physical activity they ought to accumulate based, in part, on how others in their group perform and made changes to their behavior accordingly, researchers could draw on social comparison theory [39] and test the role of social comparisons. In addition, given that the nurses’ working environment and job characteristics can have detrimental effects [59,68-70], the extent to which nurses’ workload, responsibilities, and working hours (eg, shift length and type of shift worked) influence their ability to engage in physical activity and the effectiveness of physical activity interventions should be studied. Finally, as increases in physical activity levels were not maintained over the course of the intervention, further research is clearly warranted to determine how web-based worksite interventions for nurses can be improved to support long-term changes in physical activity. On the basis of previous research [30,71-75], providing (1) individually tailored lifestyle advice, (2) physical activity plans and targets, (3) information on the benefits of physical activity, (4) physical activity self-monitoring devices, (5) interactive lectures, (6) weekly aerobic exercise classes, and/or (7) short exercise breaks at work should be considered. Furthermore, integrating behavior change techniques, implementing cognitive behavioral training, and manipulating the worksite may increase the effectiveness of the intervention [64,76]. Thus, future research to assess the effectiveness of physical activity interventions should be (1) tailored to the nurses’ individual needs, (2) address macro-level changes (ie, policy changes and environmental modifications), and (3) designed, implemented, and evaluated based on theory.

### Conclusions

Although the International Council of Nurses has called for nurses to make “a personal commitment to eat healthily, exercise appropriately, drink sensibly and avoid the use of tobacco” [77] and the growing expectation that nurses should embody those behaviors they wish to promote [78], most nurses report low physical activity levels [14,79,80]. Despite this, worksite interventions aimed at increasing physical activity levels among nurses are scarce. Moreover, little consideration has been given to factors that may predict changes in physical activity levels within an intervention. The principal conclusion of this study is that external and identified regulations for physical activity predicted changes in objectively measured physical activity levels. Accordingly, strategies to promote motivation for physical activity, external and integrated regulations in particular, should be part of larger strategies to promote physical activity in future interventions. Nevertheless, as initial increases were not maintained over time, the findings also highlight that changing nurses’ long-term physical activity behavior is difficult and requires continued effort. Work-related circumstances (eg, job strain, nurse shortages, workload, long hours, and night or irregular shifts) may introduce barriers (eg, fatigue and lack of time) for physical activity. It is necessary to continue to investigate both personal and occupational factors that could help the nurses sustain physical activity levels in the long term. Using qualitative methods (eg, in-depth interviews, focus group discussions, and observations) may aid in the understanding of such factors and provide insight into what the nurses themselves thought of the intervention. Finally, key stakeholders should be involved in the development, implementation, and evaluation of future worksite physical activity interventions for nurses to ensure they are feasible, sustainable, and adaptable to specific workplace demands.

## Appendix

Multimedia Appendix 1 Online Tractivity® program which displayed participants distance, steps, active time and calories expended on an hourly, daily, weekly, and monthly basis.

Multimedia Appendix 2 Friend challenge in online Tractivity® program which displayed the total distance and steps of another participant randomized to the friend challenge.

Multimedia Appendix 3 Team challenge in online Tractivity® program which displayed the total distance and steps of others teams randomized to the team challenge.

## Abbreviations

BREQ-2: Behavioral Regulation in Exercise Questionnaire-2
MVPA: moderate-to-vigorous physical activity
