# Supplementary material for: Motivation Predicts Change in Nurses’ Physical Activity Levels During a Web-Based Worksite Intervention: Results From a Randomized Trial
Source: J Med Internet Res. 2020 Sep 11;22(9):e11543. doi: 10.2196/11543 (PMC7519423; doi:10.2196/11543)
Supplement: Multimedia Appendix 1 [file jmir_v22i9e11543_app1.pdf]

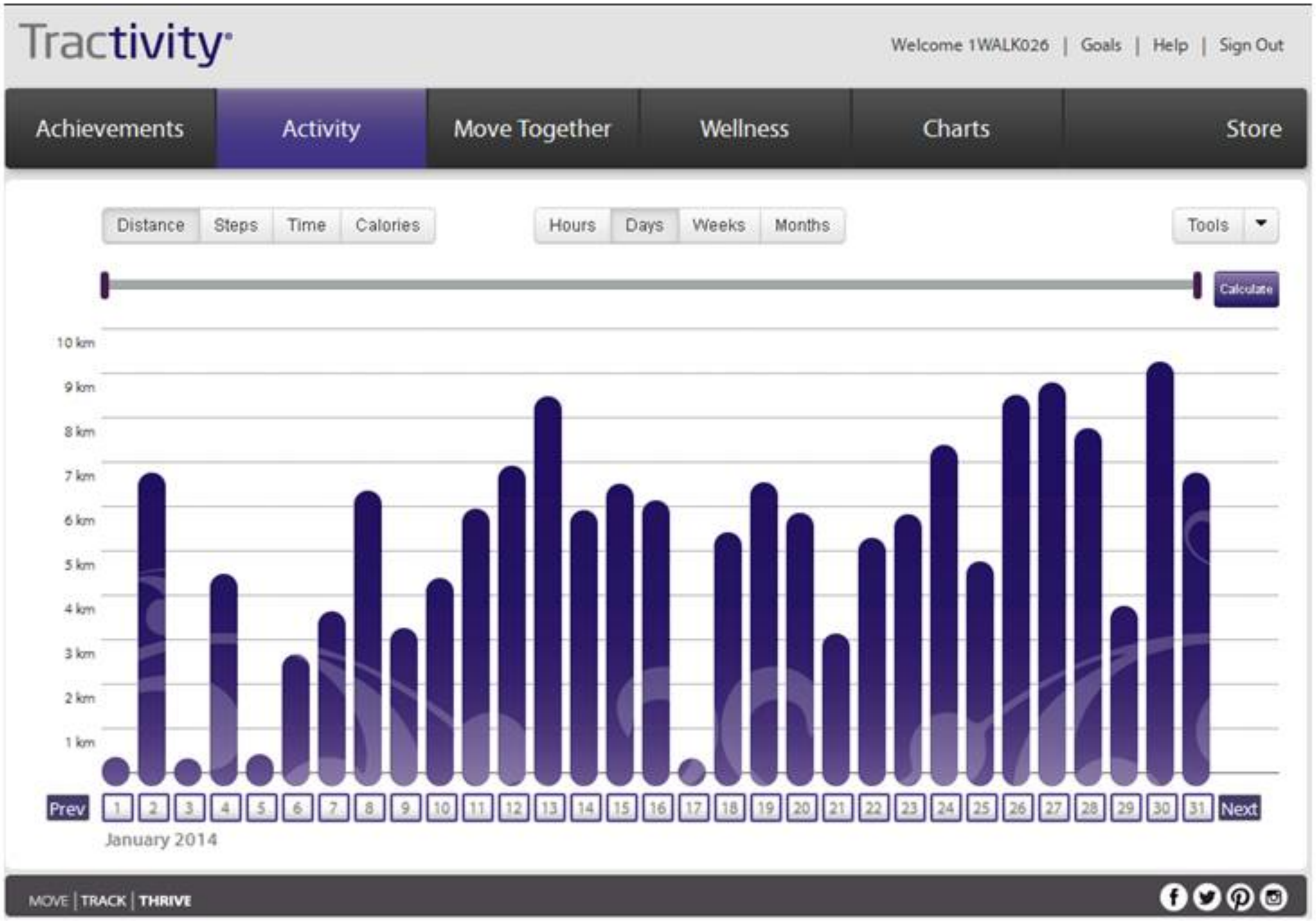

Tractivity
Welcome 1WALK026 | Goals | Help | Sign Out
Achievements
Activity
Move Together
Wellness
Charts
Store
Distance
Steps
Time
Calories
Hours
Days
Weeks
Months
Tools
Calculate
10 km
9 km
8 km
7 km
6 km
5 km
4 km
3 km
2 km
1 km
Prev
1 2 3 4 5 6 7 8 9 10 11 12 13 14 15 16 17 18 19 20 21 22 23 24 25 26 27 28 29 30 31
Next
January 2014
MOVE | TRACK | THRIVE
