# Supplementary material for: Motivation Predicts Change in Nurses’ Physical Activity Levels During a Web-Based Worksite Intervention: Results From a Randomized Trial
Source: J Med Internet Res. 2020 Sep 11;22(9):e11543. doi: 10.2196/11543 (PMC7519423; doi:10.2196/11543)
Supplement: Multimedia Appendix 3 [file jmir_v22i9e11543_app3.pdf]

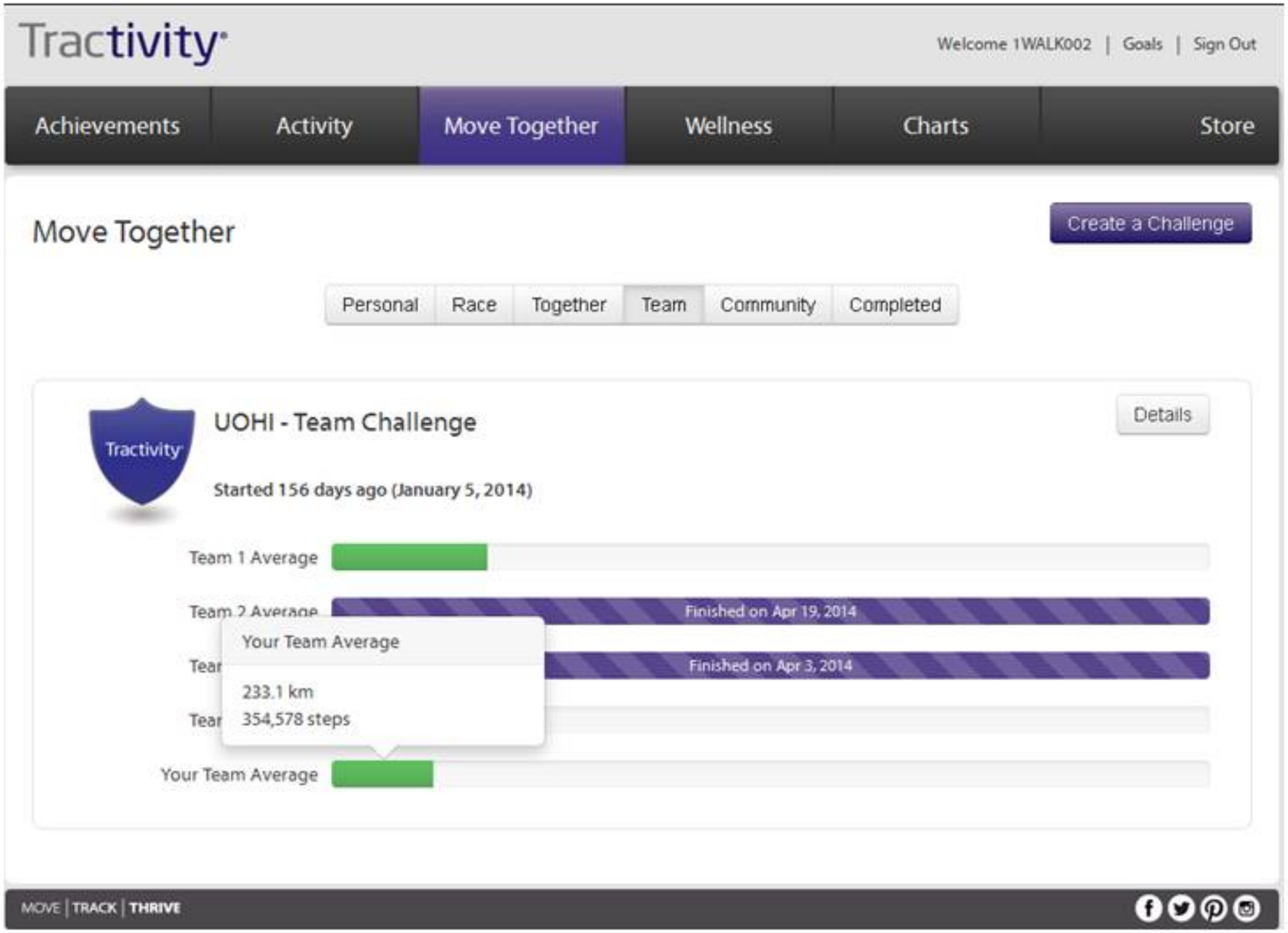
Tractivity
Welcome 1WALK002 | Goals | Sign Out
Achievements
Activity
Move Together
Wellness
Charts
Store
Move Together
Create a Challenge
Personal
Race
Together
Team
Community
Completed
Tractivity
UOHI - Team Challenge
Details
Started 156 days ago (January 5, 2014)
Team 1 Average
Team 2 Average
Finished on Apr 19, 2014
Your Team Average
233.1 km
354,578 steps
Finished on Apr 3, 2014
Your Team Average
MOVE | TRACK | THRIVE
